# Does literature evolve one funeral at a time?

**DOI:** 10.1098/rspb.2024.2033

**Published:** 2025-02-05

**Authors:** Oleg Sobchuk, Bret Beheim

**Affiliations:** ^1^ Department of Human Behavior, Ecology and Culture, Max Planck Institute for Evolutionary Anthropology, Leipzig, Germany

**Keywords:** cultural evolution, literary fiction, decomposition, cohorts, Price equation

## Abstract

The cultural evolution of literary fiction is rarely studied, but rich literary data can help address some of the general problems of cultural change. In this article, we use a massive dataset of Anglophone fiction (over 23 000 books) and the tools of natural language processing to understand whether the long-term change of topics in books is driven by the individual change of authors or by the cohort turnover in author populations. To answer this question, we borrow a method from evolutionary ecology: decomposition analysis based on the Price equation. To prove the suitability of this method, we first apply it to simulated data and show that it does allow distinguishing between these two processes. Afterwards, we decompose the temporal trajectories of topics and measure the relative effects of the arrival of newcomer authors (entrances), the retirement of authors (exits) and the change of topic preferences during authors’ lifetimes (individual change). We find that cohort turnover is a stronger force than individual change. Within the cohort effects, the effect of entrances is almost twice as strong as the effect of exits. Using simulated data, we discover that this difference stems from the unequal lengths of authors’ careers.

## 1. Introduction

Max Planck, the author of a pivotal discovery in physics, Planck’s constant, was also an author of a surprisingly pessimistic view of scientific progress, known as Planck’s principle. According to the German physicist, ‘[a] new scientific truth does not triumph by convincing its opponents and making them see the light, but rather because its opponents eventually die, and a new generation grows up that is familiar with it’ [1]. This view of scientific change is sometimes expressed briefly: ‘science advances one funeral at a time’ [2]. Max Planck made this observation about science, but science is not the only creative domain with visible intergenerational tensions. Young musicians, who want to suggest new music genres, have to compete with the already established musicians; young writers, who conceive new styles of writing, must overcome older ways of writing and so on. Generations compete in all areas of culture, but does this competition really get resolved in the sombre way envisioned by Max Planck?

In this article, we will test Planck’s principle on literary fiction—aiming to understand whether the topics in literature change ‘one funeral at a time’. The choice of literature as a test case may be unexpected, but there is rationale behind it. Science—like many other creative areas—increasingly becomes a ‘team sport’ with big collectives behind each article [3,4]. And so, the contributions of individuals—and their age cohorts—are hard to parse out. Book writing, on the other hand, has traditionally been a solitary activity (even though editors and occasional ghostwriters play a role too), which makes literature a convenient subject for studying the connection between creativity and age cohorts. Literary scholars paid attention to writers’ cohorts too: say, to the ‘lost generation’ of writers of the 1930s or the ‘beat generation’ that entered the scene in the 1950s. Moreover, a famous and controversial hypothesis states that newcomer writers have the ‘anxiety of influence’ towards their predecessors and thus are actively trying to differentiate themselves from previous generations [5].

To test Planck’s hypothesis on literature, we will use a massive dataset of novels: a subset of the HathiTrust Digital Library with 23 162 works by 6177 authors, published during 1850−1940. To analyse these data, we will combine two proven methods: topic modelling and decomposition analysis. While topic modelling with latent Dirichlet allocation (LDA) is a standard technique in digital humanities and natural language processing [6,7], decomposition analysis based on the Price equation is a standard tool in evolutionary ecology [8,9]. In ecology, decomposition analysis allows ‘dissecting’ historical trajectories of phenotypic traits of an organism (say, their size) into underlying environmental factors (say, temperature change or the changing number of predators). In this study, we will use the frequencies of LDA topics in each book as their ‘phenotypic’ traits, and demographic data of authors as ‘environmental’ factors.

## 2. ‘Generally mixed’ evidence

Intuitively, Planck’s principle may sound plausible, and many scholars just assumed its correctness based on anecdotal evidence, as did Thomas Kuhn in *The Structure of Scientific Revolutions* [10]. But what do empirical studies show? In a comprehensive review of quantitative studies, Jones *et al*. conclude that ‘evidence for Planck’s principle is more generally mixed’ [11]. Some scholars have found support for it. Azoulay *et al*. found that the deaths of key scientists led to collapses of their subfields [2]; see also [12]. The scholars of digital humanities discovered that cohort turnover seems to explain more variation than individual change in literary fiction [13,14]. In the arts, newcomer painters were found to be the ones bringing new perspectives to portraits [15]. At the same time, some studies reached the opposite conclusion about Planck’s principle. For example, an elegant quantitative study of the adoption of the tectonic plate theory shows that no generational turnover was necessary for the theory to become widely accepted [16]. Even more interestingly, older scholars were more eager to adopt the new theory than younger scholars—arguably because they had better job security and thus better position to accept unorthodox views. Similar findings are reported in a number of other articles [17,18].

Why are these findings contradictory? Part of the reason, we think, is that the operationalizing of demographic forces differed from study to study. Some papers define cohorts only as the arrival of new individuals, while others are focused on their exits (deaths, retirements). In reality though, cohort turnover is a composite process that includes *both* the ‘entrances’ (births, or rather career beginnings) and ‘exits’ of individuals (either literal deaths or retirements). Another reason is the inability of the methods used in these articles to capture the temporal dimension of the cohort effects. Cohort effects are not timeless: some periods of the history of science or arts can be more favourable to newcomer individuals than other periods. Physicists in the first half of the twentieth century were often making discoveries at a young age (think of Albert Einstein, Werner Heisenberg or Paul Dirac), but this could be due to particular historical circumstances of that period. To solve the problem posed by Max Planck more than 75 years ago, we need an approach that (i) allows to effectively decompose the frequency change of traits into cohort turnover (further subdivided into births/entrances and deaths/exits) and individual change, as well as having (ii) the temporal granularity to capture the change in these effects over time. We suggest that the decomposition analysis is fit for this task.

In what follows, we will first explain the steps of decomposition analysis. Then, we will show that this method does the given task correctly, on simulated data. Next, we will explain the steps of preparing actual literary data for the analysis. Finally, we will conduct the analysis and answer Planck’s question.

## 3. Decomposition analysis and the decomposition plot

George Price was the first to recognize that the change in a trait’s frequency over time could be partitioned exactly into the sum of multiple covariance and expectation terms representing distinct evolutionary processes, now famously known as the ‘Price equation’ in evolutionary biology [19]. Formally, if the average of some phenotypic variable is $\overset{-}{\phi }$, define $\Delta \overset{-}{\phi }$ as the *change* in this average between two time points, eras or generations. In abstract form, the Price equation states that


(3.1) $$\overset{-}{w}\Delta \overset{-}{\phi }=cov\left(w,\phi \right)\;+\;E\left(w\delta \right).$$

That is, the change in the population average of some individual-level trait $\Delta \overset{-}{\phi }$ is equal to the covariance between individual traits ϕ, and individual fitness values w, plus the average change of the trait within each lineage, E(wδ). To make the equation balance correctly, the left side must be scaled in terms of the average population fitness $\overset{-}{w}$.

In the abstract form of equation (3.1), each term in the Price equation is open to substantial interpretation. The ‘fitness’, w, could mean the number of offspring an individual has in a genetic evolutionary model, the number of cultural ‘descendants’ or the individual’s own ability to persist through some adverse conditions (surviving a harsh winter or staying employed in a trade) over time. The variable ϕ is often used to represent both gene frequencies or phenotypic measures such as body weight but may also represent some measurable behaviour or cultural characteristic that is a property of an individual [20]. Similarly, the δ term might represent the average difference between parents and offspring, or the measurable growth and development taking place within an individual through time.

The Price equation has been used for studying culture, but so far it has mostly featured in the debates about general theoretical problems of cultural evolution—usually in the context of theoretical discussions of simulations [21–23]. In evolutionary demography, a different approach was taken, by incorporating explicit, measurable processes such as immigration, growth, mortality and reproduction into the terms of the Price equation. As a result, we can decompose the trajectory of a particular cultural trait over time into terms that are both measurable and meaningful [20].

Here, we apply these general ideas to a population of fiction writers, whose primary ‘fitness’ measure is their continued contributions to the literary world. In equivalent terms as above, we might write:


(3.2) $$R\Delta \overset{-}{\phi }=\;-cov\left(e,\phi \right)+r\overset{-}{\delta },$$

where ϕ now measures the frequency at which an author employs a particular topic in their work in a given period, and r is the rate at which writers *remain* active in the community between two time periods (with e=(1-r) representing the rate at which they leave the population). As above, the average change in the frequency of the topic within a group of writers between two time periods *must* equal the sum of the covariance between the attrition of writers and the extent to which they use that topic, and the average change within surviving writers between time periods. As in equation (3.1), the equation balances with the inclusion of overall growth rate R.

Although this equation is correct for a closed cohort of writers through time, it does not accurately account for the role of *new* writers as they appear on the scene. If new arrivals ‘enter’ a population between two periods of time, the full calculation becomes:


(3.3) $$R\Delta \overset{-}{\phi }=cov\left(i,\phi \right)-cov\left(e,\phi \right)+r\overset{-}{\delta },$$

for some immigration rate i. From this, we can define the ‘entrance effect’ as the magnitude of the first covariance, $cov\left(i,\phi \right)$. In other words, if new authors tend to enter the literary world employing a particular topic in their work above average, there is a *positive* covariance, and so the average will increase. Conversely, if new authors tend not to employ a topic very often compared with the existing authors, the average will decline. Thus, the effect can either be positive or negative on the overall frequency of some trait. The same is true for the ‘exit effect’, defined here as the second covariance term, $cov\left(e,\phi \right)$, and also for the importance of individual authors changing their own topical focus in their writing as the final term, $r\overset{-}{\delta }$.

**Figure 1.**
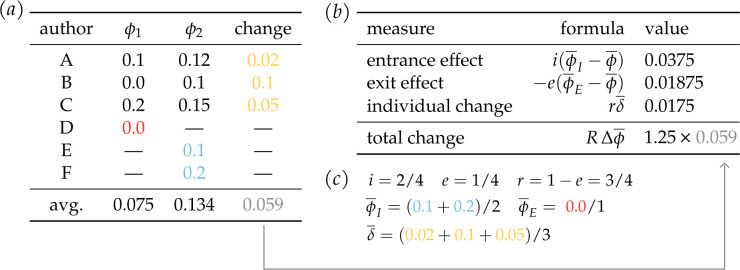
A minimum working example of decomposition showing six authors (A–F) under two periods of observation. (***a***) Authors A, B and C are active in both periods: they are writing about topic ϕ with frequencies given by column $\phi_{1}$ for period 1 and $\phi_{2}$ for period 2. The change in each author’s topic frequency is given in the rightmost column. Author D retires at the end of period 1, and so is not present in period 2 and has no ‘change’ to be observed. Authors E and F join the population in period 2, and so are observed only once as well. The average trait frequencies and the average change in trait frequency are given at the bottom. Panels (***b*** and *c*) show the calculated effects from the data given in panel (***a***) using equation (3.4). The entrance effect measures the contributions of authors E and F to the average change in mean, the exit effect measures the contribution of author D’s departure, and the average individual change is given for authors A, B and C. *R* stands for the rate of change in the number of authors from period 1 to period 2 (*R* = 5/4).

In contrast to equation (3.1), this expression has the advantage of being readily computable from real data, in the equivalent form:


(3.4) $$R\Delta \overset{-}{\phi }=i\left({\overset{-}{\phi }}_{I}-\overset{-}{\phi }\right)-e\left({\overset{-}{\phi }}_{E}-\;\overset{-}{\phi }\right)+r\overset{-}{\delta },$$

where each term represents an average rate or an average trait value for some set of individuals being tracked (figure 1). In each case, the size and magnitude of the effect can be computed from empirical data and, through the properties of covariance mathematics, will *necessarily* provide the overall trajectory of that topic, either increasing, decreasing or staying the same.

**Figure 2.**
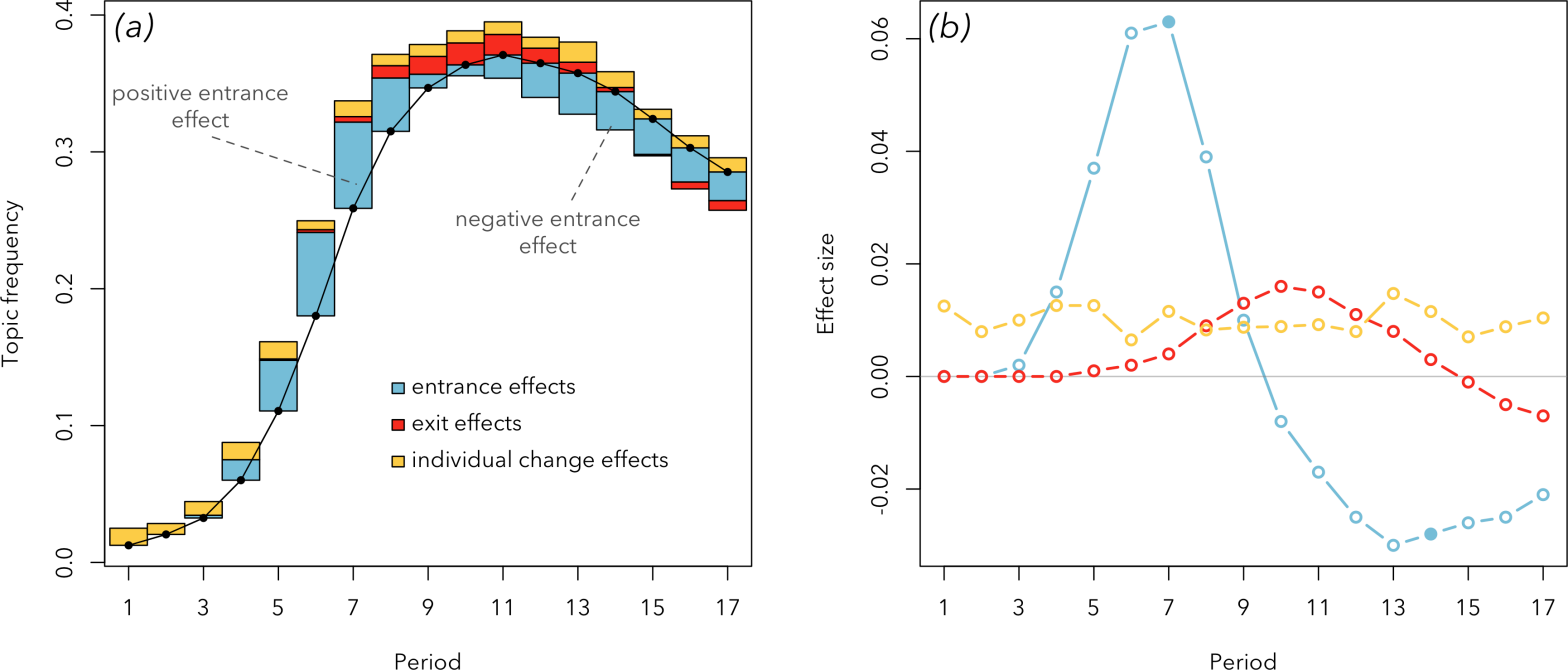
Two alternative visualizations of the decomposed effects of a single hypothetical trait (e.g. a topic). (*a*) The ‘decomposition plot’: a new kind of visualization that we suggest using. The thicker black line shows the frequency of a trait over time; coloured bars show relative sizes of effects explaining the dynamics of that trait. In this hypothetical case, the topic increases in frequency due to sustained positive effects of entrances into the population, representing a new cohort of individuals who bring this topic in with them, with a smaller positive effect due to systematic adoption of the topic (individual change). As time passes, older individuals lacking the topic leave the population (an exit effect), causing the trait to increase further. In the fullness of time, though, new cohorts begin arriving who do not have the topic, causing the entrance effect to reverse in size, stopping the growth of the topic’s popularity. The steady decline is due to both negative entrance and exit effects. To better grasp the meanings of effects, see table 1. (*b*) The line plot commonly used to visualize decomposed effects; on it, each line shows the sizes of separate effects over time. The two entrance effects corresponding to the written labels in (*a*) are given as filled circles.

To visualize the decomposed effects, we use an uncommon, but useful, visualization, which we call the ‘decomposition plot’ (figure 2*a*), introduced in Beheim & Baldini [20]. We use it instead of the line plot commonly employed for visualizing decomposed effects [8,9], shown in figure 2*b*. In the line plot, each line means the size and direction (positive or negative) of each effect at a given period of time. For example, we can see that the first half of the observed timespan is dominated by a strong positive entrance effect; the second half is driven by the positive exit effect and negative entrance effect. Our suggested decomposition plot shows the same information but has one crucial difference: instead of placing the effects on the abstract horizontal line, it places them on the empirical line showing the actual frequency of the trait (e.g. a topic) over time.

Why introduce this new visualization? First, the decomposition plot allows capturing an important additional aspect of information: it is often important to know the shape of the trait dynamics to understand the decomposed effects, as we will show later in the article. Second, it captures more intuitively the very idea of decomposition: the actual temporal dynamics of a trait (the black line on figure 2*a*) gets sliced into layers of factors that help understand these dynamics: the coloured bars. How shall one understand these bars? Table 1 explains the meanings of effects used in our study (in principle, the decomposition analysis can include many other effects).

**Table 1.** The explanation of decomposed effects shown in figure 2.

effect	direction	meaning
entrance effect	positive	newcomer authors are writing on this topic.
entrance effect	negative	newcomer authors are *not* writing on this topic.
individual change effect	positive	the authors switch *to* writing on this topic sometime during their career.
individual change effect	negative	the authors switch *away* from writing on this topic sometime during their career.
exit effect	positive	retiring authors are *not* writing on this topic.
exit effect	negative	retiring authors are writing on this topic.

While decomposition analysis is a powerful tool for finding possible forces behind the trendlines, it has its limits. We included three factors in our decomposition analysis—entrances, individual change and exits—but in principle their number is endless. The factors chosen by us have some obvious importance, and their role for literary change is easy to justify. However, it is possible to add terms to this equation that will not be so easily justifiable. The same as with any other statistical analysis, the mindless addition of terms to the equation may lead to finding spurious correlations: something that was criticized in ecology with relation to using overly abstract, unintuitive variables in the Price equation [24].

## 4. Decomposing simulated careers

Does decomposition analysis actually do what we expect it to do? To test whether this method can distinguish between different generative processes, we use stochastic simulations. We repeatedly simulate a dynamic population of 10 000 authors with realistic career lengths. Author careers begin randomly between simulation years 1850 and 2000, and their subsequent career lengths follow a Gompertz distribution with *η* = 1/20 and *b* = 1/17, which gives biologically realistic distributions of human lifespans of 63 ± 17 years [25]. These assumptions about career start and span lead to a stable population size of roughly 3000 active authors for any year between 1925 and 2000.

Authors in the simulation can produce their first work at the age of 19 years old, and subsequent works by a specific author are generated across their lifespan by a Poisson process, which produces stochastic event times. To reflect variability in author productivity, each author is randomly assigned a different lifetime productivity rate (*λ*) via an exponential distribution with an average rate of one work per decade. Together with Gompertz-distributed career lengths, these individual rates give the total number of works produced by each author within their lifetime. By the properties of the Poisson process, these works are then uniformly distributed across their career [26].

**Figure 3.**
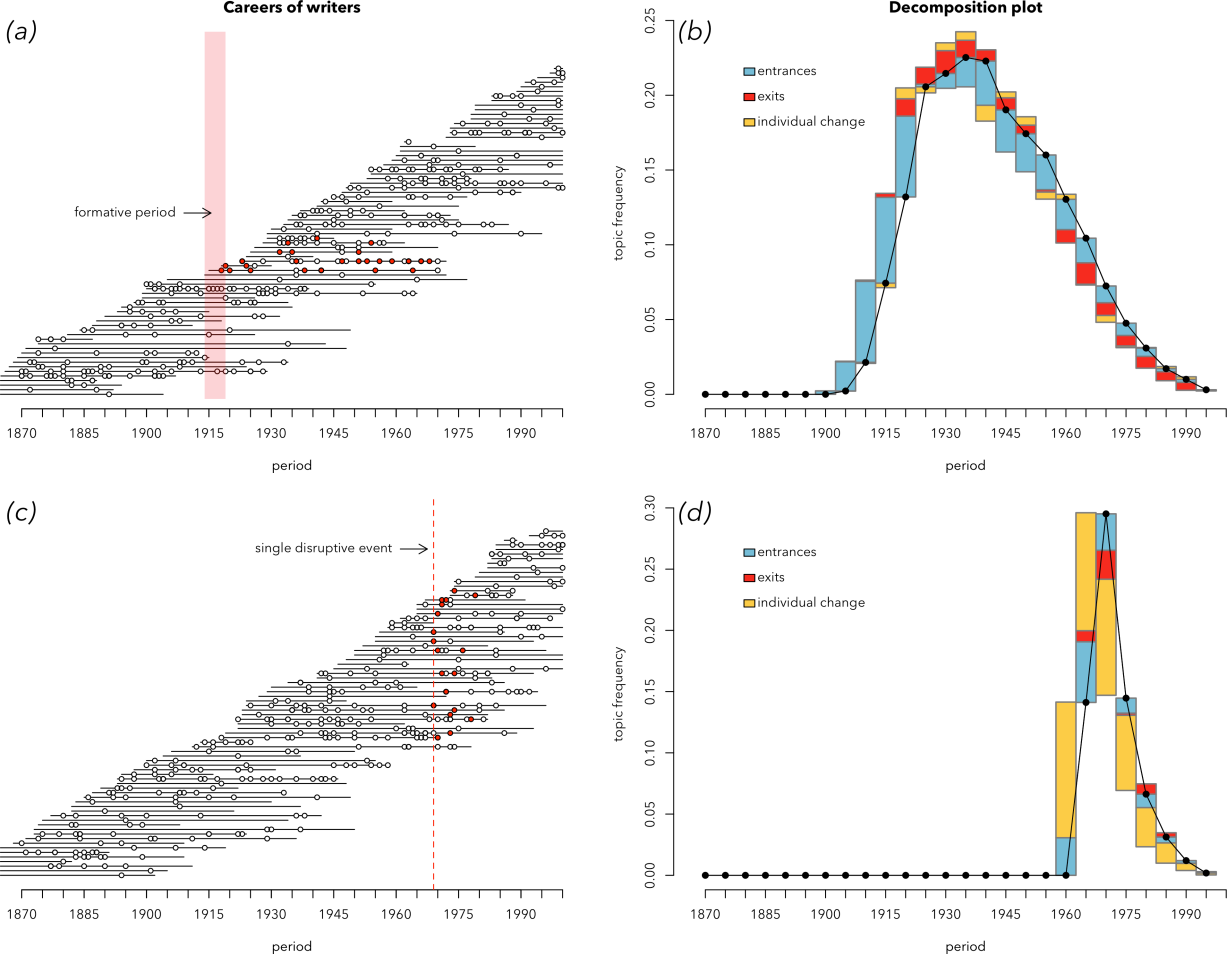
Career trajectories simulated according to the model of cohort turnover (*a*) and the model of a disruptive event (*c*). Each horizontal line represents an individual’s lifespan, and the dots—works produced by that individual during their lifespan (let’s say, books). Coloured dots are books containing a particular trait of interest (e.g. a given topic). In the case of the disruptive-event model, a particular event, such as a war (red vertical line) makes authors switch to writing on this topic, no matter at which career stage they are. In the case of the cohort model, authors born around a certain date are writing about topic *x* throughout their lifetime. The decomposition plots on the right show these two simulated datasets decomposed with the help of decomposition equations. Panel (*b*) shows that the growth of a cohort-based trait is driven by the positive effect of entrances and the positive effect of exits, while the decline of this trait is driven by the same two effects, with reversed signs: the negative entrance effect and the negative exit effect. Panel (*d*) shows that the disruptive-event simulation is driven, as expected, by the effect of individual change: at first, as the trait is growing in popularity, a positive effect of individual change, and then—a negative one.

Having defined the timings of each work within each author’s lifespan, we then simulate whether a certain topic is or is not present in any specific work via a number of distinct generative processes. In a *single-cohort* dynamic, individual authors are most likely to use a topic consistently throughout their career if they begin their careers around a specific year (figure 3*a*). In a *disruptive-event* dynamic, works are most likely to include a particular topic if they are produced in a specific year, with exponentially decaying chances in subsequent years (figure 3*c*). Additional processes are described in the electronic supplementary material (see figures S11–15).

## 5. From raw texts to a period-author matrix

**Figure 4.**
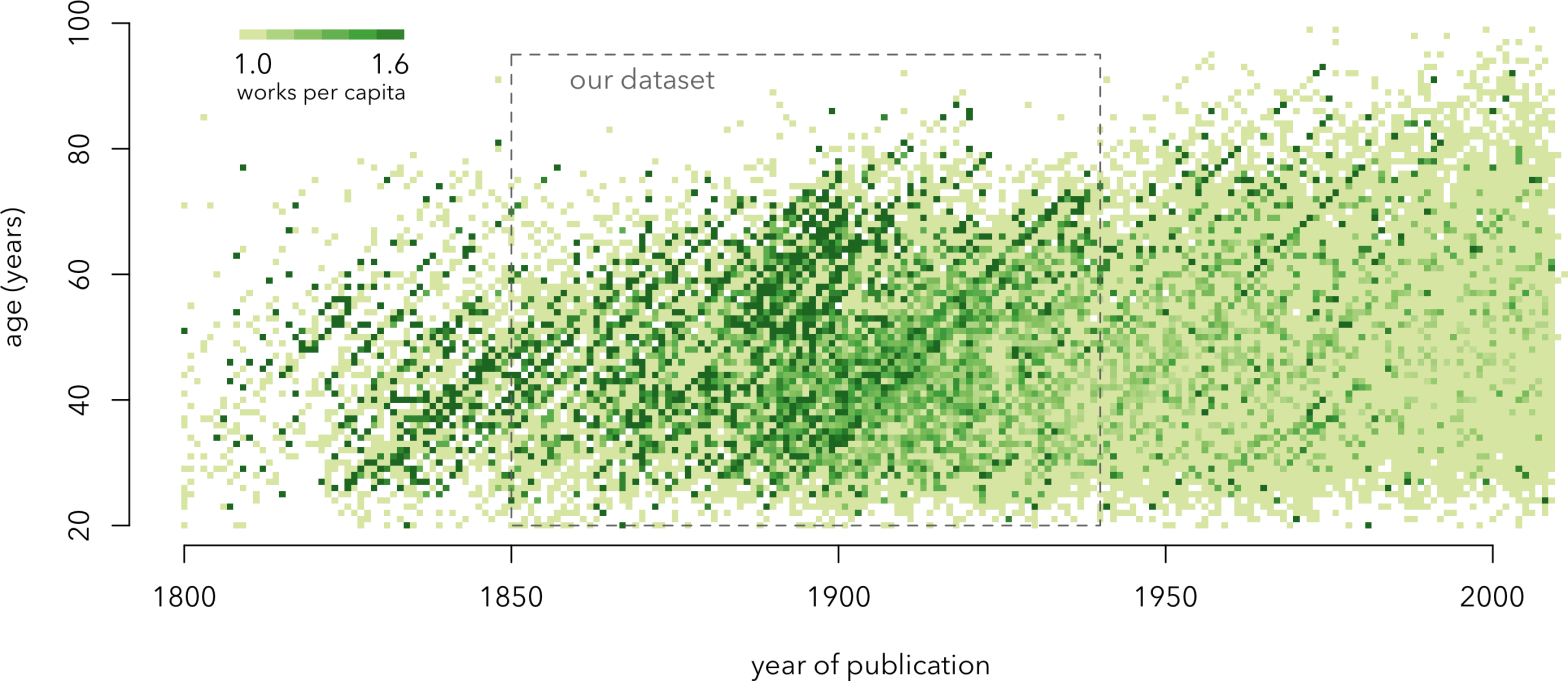
Lexis plot with time of a book’s publication on the horizontal axis and the age of an author at the time of publishing that book on the vertical axis. The intensity of colour shows the number of books published in a given year by a given set of writers of the same age. This plot makes immediately visible two properties of our data. The left side of the plot has many empty cells, probably due to the data preservation problem: the works of many authors have not been digitized. The right side of the plot does not have many empty cells, but it has many light-coloured cells, pointing at another issue: in the more recent part of the data, authors are represented by a few books each. The most complete data—with few empty cells and few light cells—lies in the middle of the plot. This is the subset we are using for analysis.

For our data, we will use HathiTrust: a massive digital library with full texts of books. We will use a subset of these books—Novel TM dataset of anglophone literature [27]. Several aspects make HathiTrust’s Novel TM stand out compared with other datasets of books. First, it includes not only the public domain books but also the ones that are under copyright. To make this possible for research purposes, HathiTrust provides access to ‘scrambled’ texts—readable for machines but unreadable for humans. These are page-wise lists of words with their frequencies. Second, Novel TM corpus includes metadata necessary for cohort analysis: birth and death years of writers and the years of first publication of their works. For our study, we decided to use only fictional works published between 1850 and 1940. The reason for this choice is shown in figure 4: the chosen period of time has the highest density of data, thus being more likely to be complete.

We performed multiple steps to clean the data. First, we removed all the books that are unlikely to be fictional (Novel TM’s non-fiction probability of more than 0.4). Also, we removed all writers with absent or uncertain birth and death years. We removed all the books that were not first publications (e.g. collected works) and removed occasional duplicate books. In the end, our corpus included 23 162 works by 6177 authors.

Decomposition analysis requires representing individuals analysed as sequences of traits. What would be the traits of literary texts for such an analysis? A long tradition of research in the humanities has been claiming that narrative ‘motifs’—recurring events and themes—are useful for studying the evolution of narratives: both for simple stories such as folk tales [28,29] and for complex narratives such as modern fiction [30]. For example, J.K. Rowling’s *Harry Potter and the Philosopher’s Stone* and Umberto Eco’s *The Name of the Rose* both share the motif of investigating a mystery, while also not sharing various motifs: Rowling’s book has a fantasy setting, while Eco’s classic is set in the Middle Ages. LDA topic modelling is suitable for capturing similar thematic motifs, since it represents each text as a combination of ‘topics’ and each topic has a certain probability to be featured in that book (see electronic supplementary material, figure S6). LDA topics are not capable of capturing specific narrative events, as manually coded motifs do, but they are well suited for capturing more abstract recurring themes. Newer methods of topic modelling such as BERTopic or Top2vec [31,32], while arguably having marginally better performance [33,34], do not allow representing texts as distributions of all topics—which is what we need for this study.

As often happens with historical data, our corpus is unevenly distributed—with the largest number of books per year in the early 1900s. When doing topic modelling of such uneven distribution over time, topics will largely be based on time periods with more material. To counter this data preservation bias, we decided to construct topic models using a uniform sample of our corpus (see, electronic supplementary material, figure S5). We made a uniform sample with 412 books per 5-year period: in total, it has 7416 books by 3083 authors. Afterwards, LDA models built on this uniform corpus were fit to the whole corpus.

We performed the following pre-processing steps. In HathiTrust corpus, each book is divided into pages, as in original scanned books. We removed three pages from the beginning and the end of each book—to get rid of occasional non-fictional parts (e.g. a foreword in the book’s beginning or an advertisement at the book’s end). We then used part-of-speech tags to filter out all the parts of language that do not convey much semantic information; we kept only nouns, verbs, adjectives and adverbs. This is a proven strategy of lowering computational costs [35] and improving recognition of genre-relevant topics [34]. We then removed 200 most frequent words, because very common words—mostly, function words (‘a’, ‘the’, ‘and’, etc.)—carry strong signal of authorship: each author has a distinctive ‘fingerprint’ made out of such words [36]. Afterwards, we lemmatized the remaining words using the *textstem* R package [37]. When casting a document-term matrix, we ranked the words by frequency and applied a 5000-word cutoff. Since novels are very long texts, and thus the context of an entire novel is not very informative for the LDA algorithm, we divided each book into 5000-word chunks.

For these chunked texts, we built an LDA topic model with the *textmineR* package in R [38]. For robustness, we constructed three topic models: with 100, 200 and 300 topics. Throughout the article, we will report the 200-topic model; results for the other two, which are not significantly different, can be found in electronic supplementary material, figure S17. Hyperparameters of the reported model, which control the shapes of prior distributions of topics and words, were set to *α* = 0.1 and *β* = 0.05. However, we tested a variety of other values and concluded that they do not influence the results (see electronic supplementary material, figure S19). After training the models, we extracted the proportions of topics (*θ*) in each book chunk and then merged them within each book by finding the mean value of *θ* across all chunks of that book.

Is using LDA justified for studying temporal changes in topic frequency? This is a common approach in the analysis of literary and, broadly, cultural change [39–42]. However, sometimes a question arises: aren’t these trends affected by language change? For example, the topic of love may be expressed in the 1950s with different words than in the 1850s. This is a legitimate concern, which would be important if we were to study, say, the dynamics of public opinions using literature only as a medium (as often newspapers are used). However, we are studying something quite different: we do not use LDA topics as simple contents of books. LDA topics, when applied to fiction, capture its meaningful repeating elements: a blend of topics *per se* with formal elements, which often correlate with literary genres [43,44].

**Figure 5.**
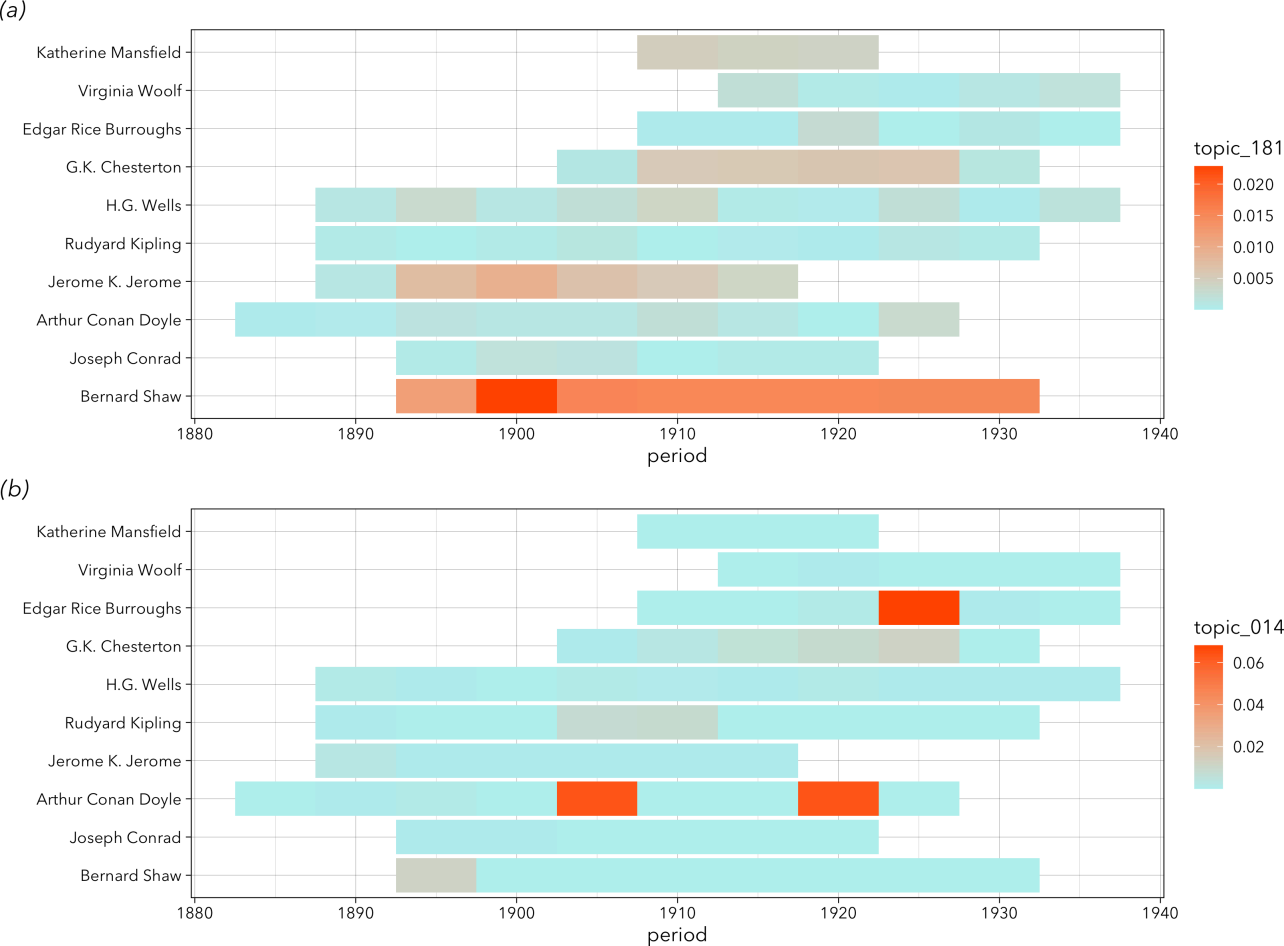
Visualization of period-author matrices, containing small samples of authors from two topics (out of 200). Rows represent careers of individual authors, divided into 5 year periods. The intensity of colour corresponds to the mean probability of a given topic across all books of this author published in this period. (*a*) A topic of stage acting (‘stage’, ‘theatre’, ‘act’, ‘actor’, ‘audience’): unsurprisingly, George Bernard Shaw, who was writing plays his whole life, has this topic highly expressed throughout his career. (*b*) Same authors, but a different topic (‘knight’, ‘castle’, ‘ride’, ‘lord’, ‘tower’). None of these authors was writing on it persistently, but some would write occasionally, such as Arthur Conan Doyle in his historical novel about the Middle Ages, *Sir Nigel* (1906).

We divided the timeline into 5 year periods, and then the data were represented as a ‘period-author matrix’. Figure 5 visualizes small samples from a period-author matrix, with two randomly chosen topics, and 10 arbitrary authors. A period-author matrix is a matrix with columns for *period*, *author_id* and multiple columns for each topic (*topic_001*, *topic_002*, etc.), with mean probabilities of a given topic across all books of an author published in a given period (see electronic supplementary material, table S2). In the case when an author had a period without any books in our dataset, we treated these as missing values and estimated the topics in that cell using linear interpolation with the *zoo* package in R [45]. Electronic supplementary material (figure S16) illustrates how the interpolation was made. We also removed the authors that happened to have books in only one 5 year period. After dropping them, we obtain a period-author matrix containing 18 890 books by 2526 authors.

## 6. Decomposing individual topics

**Figure 6.**
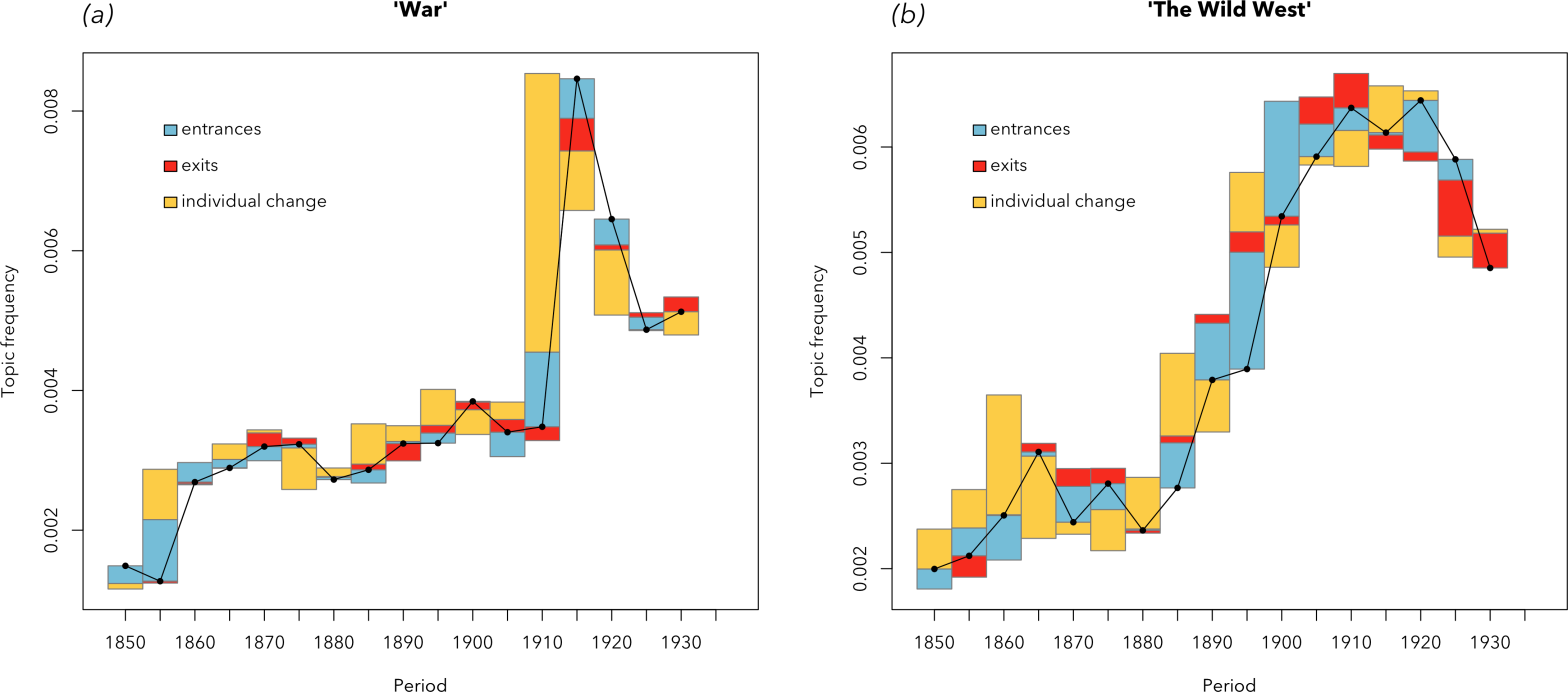
Decomposed trendlines that illustrate two scenarios, earlier demonstrated with simulations (figure 3). (*a*) A topic of war, coinciding with the First World War. It is a clear example of a change associated with an abrupt external event, when authors of various age cohorts stopped writing whatever they were writing before and began writing about this, most pressing, issue. As expected, the surge around the war is associated with the positive effect of individual change. (*b*) A topic of the Wild West, strongly associated with frontier fiction. This topic is driven by a cohort effect: its growth is associated with the entrance of many new authors (thus, positive entrance effect), and the decline coincides with the retirement of authors (negative exit effect). So, the topic was mostly used by a particular generation of writers and ceased existing when this generation retired.

The proportions of each topic in texts were separately decomposed using the decomposition equation. The results for two exemplary topics are shown in figure 6. These two topics are real-data examples of two idealized scenarios shown earlier with simulations (figure 3). Panel (*a*) shows a topic clearly associated with war (top words: ‘war’, ‘soldier’, ‘army’, ‘fight’, ‘peace’). This topic surged in frequency during 1915−1920—most likely, because of the First World War. But what was the driver behind this surge? First possibility: the war created room for a new cohort of ‘military authors’ specializing on war-related topics. Second possibility: already practicing authors, writing on various topics, pivot towards writing about war. The decomposition analysis allows us to clearly distinguish between these two possibilities. As the decomposition bars in panel (*a*) suggest, it was mostly the latter (positive individual change), and to a much lesser extent—the former (positive entrance effect). That is, writers who did not write about war-related issues before the Great War, are switching to writing about it. External shocks work like this: the recent disruptions brought by the Covid-19 pandemic or the Russian invasion of Ukraine are still fresh in our memory. Many creative individuals began writing or studying these subjects, no matter what they studied prior to that. Interestingly, we also see a moderately strong positive entrance effect around the spike: for some of these writers, their book about the disruptive event happens to be their first book.

Figure 6*b* shows a very different example: a cohort turnover. This topic is associated with books about adventures in the American Midwest (top words: ‘cabin’, ‘camp’, ‘trail’, ‘rifle’, ‘prairie’). They describe the rugged life in the wilderness, led by trappers. Often, adventure stories for kids, with telling titles: *Following the Deer* (William J. Long), *The Wolf Hunters* (James Oliver Curwood) or *The Fort in the Wilderness* (Edward Stratemeyer). The most famous author in this group was Jack London (some of the most representative books in this genre are his collections of stories). For many of these authors, the life of a trapper was their real life, or at least the life they tried at some point. And so, they did not change topics of writing often. As a result, this topic is driven by a cohort effect. The growth of this topic’s popularity happened at the turn of the century and coincided with the positive entrance effect: that is, with first publications of these authors. For example, Jack London published his first collection of stories about wildlife adventures, *The Son of Wolf*, in 1900. However, already in the 1920s, the genre started declining, and the decline is associated with the negative exit effect: that is, its decline coincides with the last books of authors. This genre is declining not because its writers switch to other topics, but because they are retiring. The genre lasted for about 40 years: a decent writing career of one generation. This generation retires, being replaced with another cohort, writing on other topics: in part, also because the trapper lifestyle no longer existed.

These are only two examples showing how, behind supposedly similar temporal trajectories, there may be hidden very different processes. We have decomposed each topic’s trajectory, many of which suggest possible explanatory mechanisms behind their dynamics (all of them are included in the electronic supplementary material). The power of the decomposition method, suggested in this article, is that it allows observing the temporal change in each individual topic across time, which was not possible with the regression-based methods used so far [14,17]. However, we are still interested in the *overall* effects across all the topics, since they will allow us to test the hunch of Max Planck.

## 7. Solving Planck’s riddle

**Figure 7.**
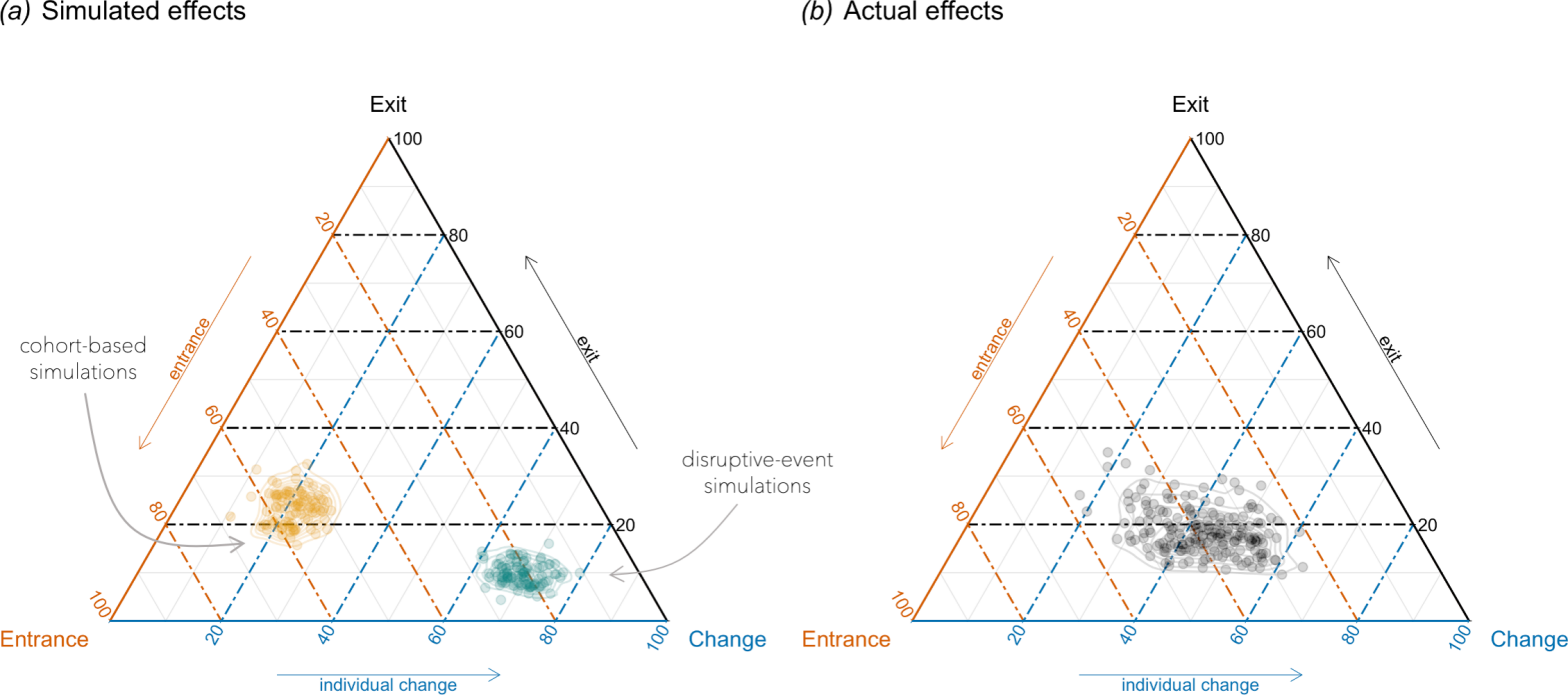
Ternary plots showing the relative weights of the effects of entrance, exit and individual change. Each point shows the effects for a single trait. (*a*) The effects for 100 simulated traits based on the *cohort* effects process (orange dots), and the effects for 100 traits simulated using the *disruptive-event* process (green dots). (*b*) Effects for the actual 200 topics in our book corpus.

What are the overall effects, across all topics? A ternary plot in figure 7*a* shows, for illustration, the effects for 200 simulated traits: 100 simulated using the cohort model and 100 simulated using the disruptive-event model. Each point shows the relative sizes of three effects in a single simulation. The closer this point is to the corner of the triangle, the stronger the ‘pull’ of that effect on the trait’s trajectory. Unsurprisingly, the traits simulated with the *cohort model* lie far from the individual change effect, being much closer to the entrance effect and less so—to the exit effect (later we will explain why this is happening). The simulations based on the *disruptive event* model, also unsurprisingly, lie close to the corner of individual change. But where are the actual effects, based on our HathiTrust dataset, located?

Figure 7*b* shows the overall distribution of the actual effects across 200 topics. Each point represents the relative weights of the three effects—entrances, exits and individual change—for a particular topic. The mean contribution of entrances in 200 topics is 38.4% (s.d. = 7.3%), of individual change is 43% (s.d. = 8.9%) and of exits is 18.5% (s.d. = 4.7%). Again, as a reminder, our method only allows us to estimate the sizes of effects compared with each other, but does not account for various other factors that may play a role, which were not included in our decomposition equation. However, the important conclusion that we can make is that the roles of entrances and individual change are roughly similar, while the role of exits is much smaller.

Our conclusion is in agreement with the results of Underwood *et al*. [14], who identified that about 55% of variation is explained with the year of birth (equivalent to our entrance effect) and 45% with individual change. They interpreted the results as evidence for a bigger role of the cohort effect. To us, however, the more important finding is that these effects are very similar (in some runs of Underwood *et al*.’s model the split was as small as 52% to 48%). This is almost identical to an even split found in our study. Still, what makes us think that cohort turnover is indeed a potent force in the change of literary topics is the effect of exits. It holds a relatively large 18.5% of the total weight of effects. If considered together, the cohort effects—the effects of entrances and exits—add up to 56.9% of the total weight.

The effects of entrances and exits—cohort effects—are larger in total than individual change. But why is the effect of entrances twofold larger than the effect of exits? The arrival of new authors and their retirement are two processes contributing to cohort turnover, mirroring each other. Should they not be *equally* large? Remember, the same happened to our simulated cohorts (figure 3*b*): the effect of entrances was noticeably larger than the effect of exits.

**Figure 8.**
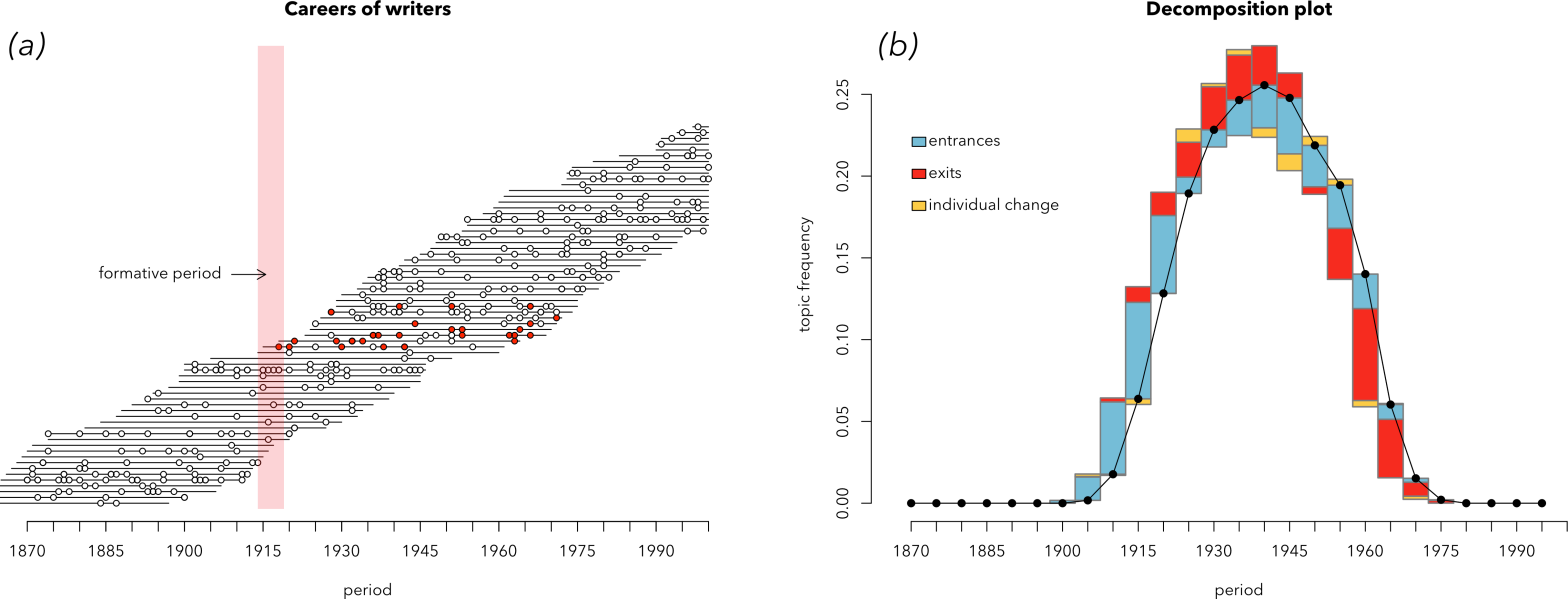
The decomposition plot of a simulated cohort-driven trait. Unlike the plot in figure 3*a*, the career spans of individuals (*a*) have equal lengths: they are drawn from a uniform distribution instead of the realistic Gompertz distribution of lifespans. Note that the effects of entrances and exits are about the same size, mirroring each other (*b*).

To solve this puzzle, we returned to simulations and discovered that the reason for the lower role of the exit effect is inequality of authors’ career spans. Previously, we simulated author careers using Gompertz distribution, which is a realistic distribution often used in demography for simulating lifespans. According to this distribution, some careers are very long, some are short and most have medium length. However, if we sample career lengths from a uniform distribution, thus making all career spans of the same length, we will end up with the situation shown in figure 8. In this case, the strength of entrances and exits is the same.

What does this mean? In the real world, where lifespans are unequal, the entrances of authors are synchronized: most authors begin publishing in their 20s. But their retirements are *not synchronized*: authors retire at different ages and for different reasons. Some authors decide that the life of a famous writer is not for them, like Salinger at the age of 46; others, never manage to achieve the desired success and die in poverty like Edgar Allan Poe at the age of 40. While almost everyone who enters the literary market happens to go through an early career stage, other stages are less predictable. And so, the collective ‘push’ of their retirement is much weaker than the collective ‘push’ of their entrance to the literary field. For example, take the female crime writers of the ‘golden age’ of crime fiction—often called the four ‘queens of crime’: Agatha Christie, Dorothy L. Sayers, Ngaio Marsh and Margery Allingham. They were all born very close to each other: in, respectively, 1890, 1893, 1895 and 1904. And they were all writing crime fiction until their deaths, which would spread across a much longer span: from 1957 to 1982. Age cohorts begin at the same time, but they end at different times.

## 8. One birth at a time

Does literature advance one funeral at a time? We have analysed a corpus of over 23 000 books by more than 6000 authors and arrived at an answer that is more nuanced than a simple ‘yes’ or ‘no’. The turnover of cohorts does play an important role in the change of literary topics, but the role of authors’ retirement is not large, being twice as small as the effect of the arrival of new authors. Literature does not advance one funeral at a time—it advances one *birth* at a time. The arrival of new authors with fresh ideas is enough to keep the wheels of literature moving. And the reason why the collective ‘push’ of the newcomers is more important than the influences of the retirees lies in the unequal career lengths.

How *generalizable* is this finding? Can we expect to find the same pattern in science, visual arts or music? In an earlier study, Beheim [46] used the decomposition approach to analyse the role of entrances, exits and individual change in the adoption of one particularly popular opening move in the game of Go, using a manually coded dataset of games. It turns out, the role of cohort turnover in Go is minimal, and the main factor is individual learning: Go players are actively changing during their careers. If we imagine that this discovery is representative of Go at large, it makes sense: in any competitive game, any player’s strategy is subject to immediate feedback. If a strategy is not working, you will be losing games, and so you will be pushed to switch to a strategy that helps you win.

Writing literature is different from playing Go: literature’s feedback mechanism is less clear and less immediate. In our period of observation (1850−1940) the main kinds of feedback the authors might be getting were critical reviews in the periodicals and book sales. However, as literary history has shown us again and again, neither negative reviews nor bad sales are good indications that a book is bad and that its author must change their approach to writing. Many writers choose to write about whatever they consider important or beautiful even if the whole world thinks otherwise.

If this reasoning is correct, science must fall somewhere between these two ends. On the one hand, it is highly competitive, and feedback loops exist. Scientists are actively trying to cross-test each other’s hypotheses and to prove each other wrong [47]—pretty much like the players in a game like Go. At the same time, many stories of scientific achievement are stories of individuals going against the opinion of the majority [48]. A large-scale study of scientific careers using decomposition analysis in the future would help us answer whether this is the case.

Another interesting question—about the temporal dynamics of these effects in culture. The Internet has offered multiple instruments for immediate feedback: view counters, ‘likes’ and so on [49]. Our prediction is that such feedback mechanisms should be changing the dynamics of the three effects included in our study: the online writers of today, who often post their content for YouTube or fanfiction websites, should be much faster at adapting to the changing landscape of trends, and so the cohort effect today must be weaker than it was before the Internet.

## Data Availability

The data and R code needed to reproduce this study can be found in a repository on Open Science Framework's website [50]. Supplementary material is available online [51].

## References

[1] Planck M. 1950 Scientific autobiography and other papers. London, UK: Williams & Norgate.

[2] Azoulay P, Fons-Rosen C, Zivin JSG. 2019 Does science advance one funeral at a time? Am. Econ. Rev. **109**, 2889–2920. (10.1257/aer.20161574) 31656315 PMC6814193

[3] Wu L, Wang D, Evans JA. 2019 Large teams develop and small teams disrupt science and technology. Nature **566**, 378–382. (10.1038/s41586-019-0941-9) 30760923

[4] Wuchty S, Jones BF, Uzzi B. 2007 The increasing dominance of teams in production of knowledge. Science **316**, 1036–1039. (10.1126/science.1136099) 17431139

[5] Bloom H. 1997 The anxiety of influence: a theory of poetry. New York, NY; Oxford, UK: Oxford University Press.

[6] Blei DM, Ng AY, Jordan MI. 2003 Latent Dirichlet allocation. J. Mach. Learn Res **3**, 993–1022. (10.5555/944919.944937)

[7] Jockers ML, Mimno D. 2013 Significant themes in 19th-century literature. Poetics **41**, 750–769. (10.1016/j.poetic.2013.08.005)

[8] Coulson T, Tuljapurkar S. 2008 The dynamics of a quantitative trait in an age‐structured population living in a variable environment. Am. Nat. **172**, 599–612. (10.1086/591693) 18840061 PMC3345271

[9] Ozgul A, Tuljapurkar S, Benton TG, Pemberton JM, Clutton-Brock TH, Coulson T. 2009 The dynamics of phenotypic change and the shrinking sheep of St. Kilda. Science **325**, 464–467. (10.1126/science.1173668) 19574350 PMC5652310

[10] Kuhn TS. 1962 The structure of scientific revolutions. (ed O Neurath). Chicago, IL: University of Chicago Press.

[11] Jones BF, Reedy EJ, Weinberg BA. 2014 Age and scientific genius. In The wiley handbook of genius, pp. 422–450. Hoboken, NJ: John Wiley & Sons. See https://onlinelibrary.wiley.com/doi/abs/10.1002/9781118367377.ch20.

[12] Packalen M, Bhattacharya J. 2019 Age and the trying out of new ideas. J. Hum. Cap. **13**, 341–373. (10.1086/703160) 31435457 PMC6703833

[13] Moretti F. 2005 Graphs, maps, trees: abstract models for literary history. London, UK; New York, NY: Verso.

[14] Underwood T, Kiley K, Shang W, Vaisey S. 2022 Cohort succession explains most change in literary culture. Sociol. Sci. **9**, 184–205. (10.15195/v9.a8)

[15] Morin O. 2013 How portraits turned their eyes upon us: visual preferences and demographic change in cultural evolution. Evol. Hum. Behav. **34**, 222–229. (10.1016/j.evolhumbehav.2013.01.004)

[16] Messeri P. 1988 Age differences in the reception of new scientific theories: the case of plate tectonics theory. Soc. Stud. Sci. **18**, 91–112. (10.1177/030631288018001004)

[17] Diamond AM. 1980 Age and the acceptance of cliometrics. J. Econ. Hist. **40**, 838–841. (10.1017/s002205070010021x)

[18] Wray KB. 2003 Is science really a young man’s game? Soc. Stud. Sci. **33**, 137–149. (10.1177/0306312703033001961)

[19] Price GR. 1970 Selection and covariance. Nature **227**, 520–521. (10.1038/227520a0) 5428476

[20] Beheim BA, Baldini R. 2012 Evolutionary decomposition and the mechanisms of cultural change. Cliodyn. **3**, 217–233. (10.21237/c7clio3212123)

[21] El Mouden C, André JB, Morin O, Nettle D. 2014 Cultural transmission and the evolution of human behaviour: a general approach based on the Price equation. J. Evol. Biol. **27**, 231–241. (10.1111/jeb.12296) 24329934

[22] Lewens T. 2023 Equations at an exhibition: on the cultural Price equation. In Evolutionary thinking across disciplines: problems and perspectives in generalized Darwinism (eds A du Crest, M Valković, A Ariew, H Desmond, P Huneman, TAC Reydon), pp. 353–373. Cham, Switzerland: Springer. (10.1007/978-3-031-33358-3_16)

[23] Nettle D. 2020 Selection, adaptation, inheritance and design in human culture: the view from the Price equation. Phil. Trans. R. Soc. B **375**, 20190358. (10.1098/rstb.2019.0358) 32146878 PMC7133501

[24] Bourrat P, Godsoe W, Pillai P, Gouhier TC, Ulrich W, Gotelli NJ, van Veelen M. 2023 What is the price of using the Price equation in ecology? Oikos **2023**, e10024. (10.1111/oik.10024)

[25] Gompertz B. 1825 On the nature of the function expressive of the law of human mortality, and on a new mode of determining the value of life contingencies. Philos Trans R Soc Lond **115**, 513–583. (10.1098/rstl.1825.0026) PMC4360127 25750242

[26] Blitzstein JK, Hwang J. 2014 Introduction to probability. Boca Raton, FL; London, UK; New York, NY: CRC Press, Taylor & Francis Group. (10.1201/9780429428357-2)

[27] Underwood T, Kimutis P, Witte J. 2020 NovelTM datasets for English-language fiction, 1700-2009. J. Cult. Anal. **5**, 13147. (10.22148/001c.13147)

[28] Propp V. 1968 Morphology of the folktale. Austin, TX: University of Texas Press.

[29] Uther HJ. 2004 The types of international folktales: a classification and bibliography, based on the system of antti aarne and stith thompson. Helsinki, Finland: Suomalainen Tiedeakatemia, Academia Scientiarum Fennica.

[30] Cawelti JG. 1976 Adventure, mystery, and romance: formula stories as art and popular culture. Chicago, IL; London, UK: University of Chicago Press.

[31] Angelov D. Top2Vec: Distributed representations of topics. arXiv. See http://arxiv.org/abs/2008.09470.

[32] Grootendorst M. 2022 BERTopic: Neural topic modeling with a class-based TF-IDF procedure. arXiv. (10.48550/arXiv.2203.05794). See http://arxiv.org/abs/2203.05794.

[33] Karjus A. 2023 Machine-assisted mixed methods: augmenting humanities and social sciences with artificial intelligence. arXiv. See http://arxiv.org/abs/2309.14379.

[34] Sobchuk O, Šeļa A. 2024 Computational thematics: comparing algorithms for clustering the genres of literary fiction. Humanit. Soc. Sci. Commun. **11**. (10.1057/s41599-024-02933-6)

[35] Maier D, Niekler A, Wiedemann G, Stoltenberg D. 2020 How document sampling and vocabulary pruning affect the results of topic models. Comput. Commun. Res. **2**, 139–152. (10.5117/ccr2020.2.001.maie)

[36] Neal T, Sundararajan K, Fatima A, Yan Y, Xiang Y, Woodard D. 2018 Surveying stylometry techniques and applications. ACM Comput. Surv. **50**, 1–36. (10.1145/3132039)

[37] Rinker T. 2017 textstem: tools for stemming and lemmatizing text. See https://cran.r-project.org/web/packages/textstem/index.html.

[38] Jones T, Doane W, Attbom M. 2015 textmineR: functions for text mining and topic modeling https://cran.rproject.org/web/packages/textmineR/index.html

[39] Barron ATJ, Huang J, Spang RL, DeDeo S. 2018 Individuals, institutions, and innovation in the debates of the French Revolution. Proc. Natl Acad. Sci. USA **115**, 4607–4612. (10.1073/pnas.1717729115) 29666239 PMC5939074

[40] Goldstone A, Underwood T. 2014 The quiet transformations of literary studies: what thirteen thousand scholars could tell us. New Lit. Hist. **45**, 359–384. (10.1353/nlh.2014.0025)

[41] Lambert B, Kontonatsios G, Mauch M, Kokkoris T, Jockers M, Ananiadou S, Leroi AM. 2020 The pace of modern culture. Nat. Hum. Behav. **4**, 352–360. (10.1038/s41562-019-0802-4) 31959923

[42] Rockmore DN, Fang C, Foti NJ, Ginsburg T, Krakauer DC. 2018 The cultural evolution of national constitutions. J. Assoc. Inf. Sci. Technol. **69**, 483–494. (10.1002/asi.23971)

[43] Schöch C. 2017 Topic modeling genre: An exploration of French Classical and Enlightenment drama. Digit. Hum. Q. **11**.

[44] Sharma A, Hu Y, Wu P, Shang W, Singhal S, Underwood T. 2020 The rise and fall of genre differentiation in English-language fiction. Proc. Workshop Comput. Humanit. Res. 97–114.

[45] Zeileis A, Grothendieck G. 2005 zoo: S3 infrastructure for regular and irregular time series. J. Stat. Softw. **14**, 1–27. (10.18637/jss.v014.i06)

[46] Beheim BA. 2012 Inferring general mechanisms of cultural evolution in observational settings. University of California, Davis.

[47] Latour B, Woolgar S. 1986 Laboratory life: the construction of scientific facts. Princeton, NJ: Princeton University Press. (10.1515/9781400820412)

[48] Wang D, Barabási AL. 2021 The science of science. Cambridge, UK: Cambridge University Press.

[49] Acerbi A. 2019 Cultural evolution in the digital age. Oxford, UK: Oxford University Press.

[50] Sobchuk O, Beheim B. 2024 Does literature evolve one funeral at a time? Open Science Foundation. See https://osf.io/uy4s7/.10.1098/rspb.2024.203339904387

[51] Sobchuk O, Beheim B. 2024 Supplementary material from: Does literature evolve one funeral at a time? Figshare. (10.6084/m9.figshare.c.7599501)39904387

